# Crystal structure of 4-({5-[(*E*)-(3,5-di­fluoro­phen­yl)diazen­yl]-2-hy­droxy­benzyl­idene}amino)-2,2,6,6-tetra­methyl­piperidin-1-ox­yl

**DOI:** 10.1107/S2056989015012049

**Published:** 2015-06-27

**Authors:** Ramazan Tatsız, Veli T. Kasumov, Tuncay Tunc, Tuncer Hökelek

**Affiliations:** a Department of Chemistry, Harran University, 63300 Osmanbey, Şanlıurfa, Turkey; b Department of Science Education, Aksaray University, 68100 Aksaray, Turkey; c Department of Physics, Hacettepe University, 06800 Beytepe, Ankara, Turkey

**Keywords:** crystal structure, spin-labeled compounds, Schiff base compounds, hydrogen bonding, π–π stacking

## Abstract

The asymmetric unit of the title compound, C_22_H_25_F_2_N_4_O_2_, contains two crystallographically independent mol­ecules. In one mol­ecule, the two benzene rings are oriented at a dihedral angle of 1.93 (10)° and in the other mol­ecule the corresponding dihedral angle is 7.19 (9)°. The piperidine rings in the two mol­ecules adopt a similar distorted chair conformation, and both have pseudo-mirror planes passing through the N—O bonds. An intra­molecular O—H⋯N hydrogen bond between the hy­droxy group and the imine N atom is observed in both mol­ecules. In the crystal, weak C—H⋯O and C—H⋯F hydrogen bonds, enclosing *R*_2_^2^(6) ring motifs, and weak π–π stacking inter­actions link the mol­ecules into a three-dimensional supra­molecular network, with centroid-to-centroid distances between the nearly parallel phenyl and benzene rings of adjacent mol­ecules of 3.975 (2) and 3.782 (2) Å.

## Abstract 2

The asymmetric unit of the title compound contains two crystallographically independent mol­ecules with the similar conformation, the piperidine rings in both mol­ecules adopt a similar distorted chair conformation and have pseudo mirror planes passing through the N—O bond.

## Chemical context   

It is well known that the 4-amino-2,2,6,6-tetra­methyl­piperidine-1-oxyl (4-amino-TEMPO) free nitroxyl radical has been attached to various organic compounds (such as aldehydes, ketons, azo compounds and carb­oxy­lic and amino acids) and biomolecules (such as lipids, proteins, steroids and metalloenzymes) (Gallez *et al.* 1992 ▸; Berliner, 1976 ▸) to yield a wide variety of TEMPO-bearing mol­ecules named as spin-labeled compounds (Rosen *et al.*, 1999 ▸; Gnewuch & Sosnovsky, 1986 ▸). These types of nitroxide free radicals have different applications such as magnetic resonance imaging (Likhtenstein *et al.*, 2008 ▸), protection from oxidative stress and irradiative damage (Hahn *et al.*, 1994 ▸), controlled ‘living’ free-radical polymerization (Hawker, 1997 ▸), spin trapping and spin-labeling in various fields of chemistry, biology and material sciences (Tretyakov & Ovcharenko, 2009 ▸). Our literature searches revealed that while a verity of TEMPO-labeled radicals with various imines, alcohol amines, carb­oxy­lic acids, salicyl­aldehydes, azo compounds, ketone derivatives have been designed, no TEMPO-labeled compound on the basis of phenyl­azo-salicyl­aldehyde compounds has been reported. We report herein the synthesis and structure of the new class title spin-labeled compound.

## Structural commentary   

**Figure 1:**
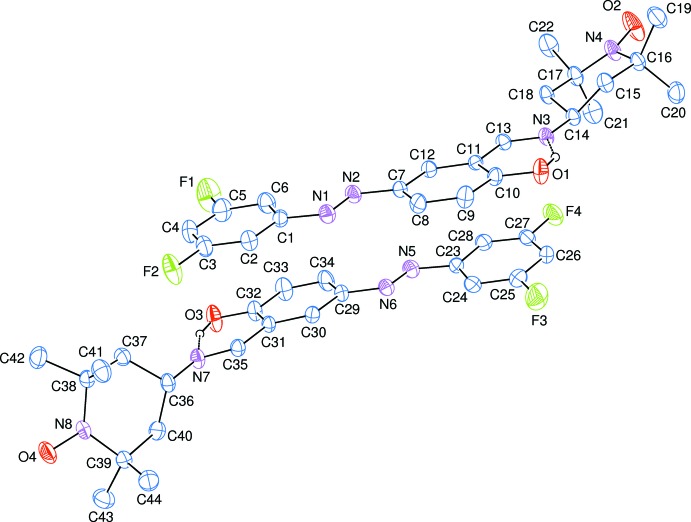
The mol­ecular structure of the title compound, showing the atom-numbering scheme. Displacement ellipsoids are drawn at the 50% probability level. Intra­molecular O—H⋯N hydrogen bonds are shown as dashed lines. C-bound H atoms have been omitted for clarity.

The asymmetric unit of the title compound contains two crystallographically independent mol­ecules (Fig. 1 ▸). The mol­ecules include short intra­molecular O—H ⋯ N hydrogen bonds (Table 1 ▸), which mean that the ligand is in the phenol–imine form. The C=N imine bond distances and C—N—C bond angles (Table 1 ▸) also indicate the existence of the phenol–imine tautomer, and they are comparable with the corresponding values of 1.276 (2), 1.279 (2) Å and 124.64 (17), 123.05 (16)° in 1,3-bis­[2-(2-hy­droxy­benzyl­idene-amino)­phen­oxy]propane (Hökelek *et al.*, 2004 ▸).
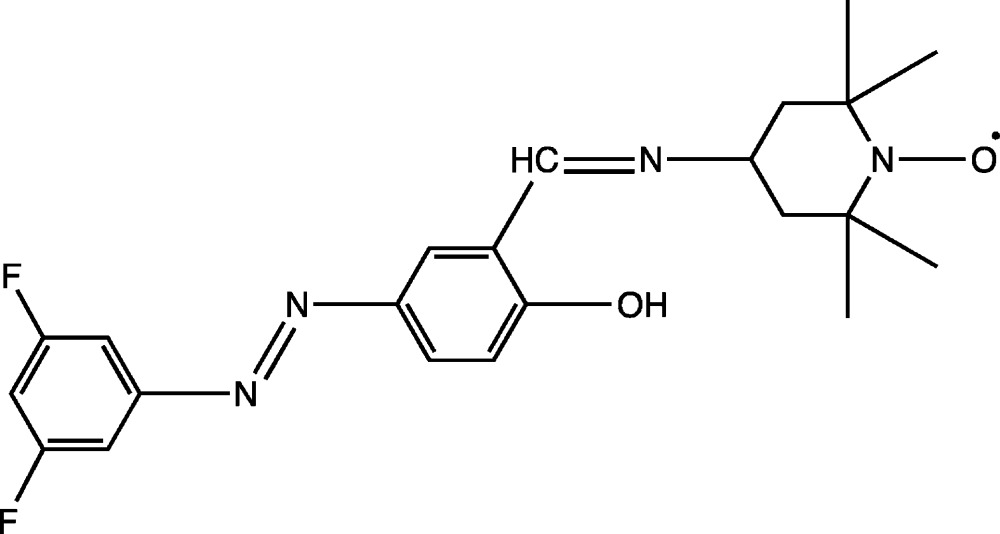


The phenyl [*A* (C1–C6) and *D* (C23–C28)] and benzene [*B* (C7–C12) and *E* (C29–C34)] rings are oriented at dihedral angles of *A*/*B* = 1.93 (10), *A*/*D* = 3.17 (10), *A*/*E* = 4.87 (10), *B*/D = 5.05 (9), *B*/*E* = 4.61 (9) and *D*/*E* = 7.19 (9)°. The six-membered rings (O1/H1/N3/C10/C11/C13) and (O3/H3/N7/C31/C32/C35) are almost planar, and they are oriented at dihedral angles of 0.83 (10) and 0.92 (9)°, respectively, to the adjacent benzene (*B* and *E*) rings.

The piperidine [*C* (N4/C14–C18) and *F* (N8/C36–C40)] rings are in distorted chair conformations [ϕ = −5.1 (9), θ = 21.7 (3)° (for ring *C*) and ϕ = −170.3 (8), θ = 157.9 (3)° (for ring *F*)] having total puckering amplitudes *Q*_T_ of 0.491 (3) Å (for ring *C*) and 0.509 (3) Å (for ring *F*), and they have pseudo mirror planes passing through the N4—O2 (for ring *C*) and N8—O4 (for ring *F*) bonds.

## Supra­molecular features   

**Figure 2:**
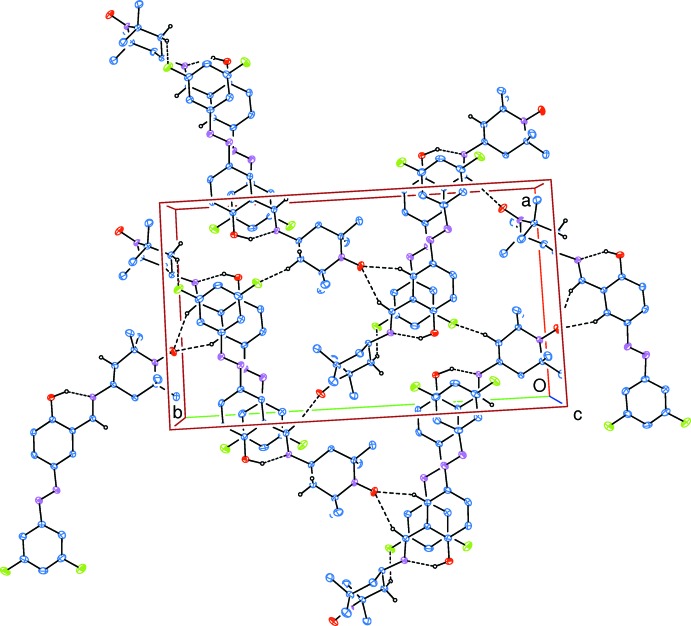
Part of the crystal structure, viewed down [001]. Intra­molecular O—H⋯N and inter­molecular C—H⋯O and C—H⋯F hydrogen bonds, which enclose 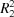
(6) ring motifs, are shown as dashed lines. H atoms not involved in these hydrogen bonds have been omitted for clarity.

In the crystal, strong intra­molecular O—H⋯N and weak inter­molecular C—H⋯O and C—H⋯F hydrogen bonds (Table 2 ▸) link the mol­ecules, enclosing 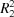
(6) ring motifs (Bernstein *et al.*, 1995 ▸) and forming layers parallel to (001), into a three-dimensional network (Fig. 2 ▸). The π–π stacking inter­actions between the phenyl and benzene rings, *Cg*1⋯*Cg*5^i^ and *Cg*2⋯*Cg*4^i^ [symmetry code: (i) *x* − 1, *y*, *z*, where *Cg*1, *Cg*2, *Cg*4 and *Cg*5 are the centroids of the rings *A* (C1–C6), *B* (C7–C12), *D* (C23–C28) and *E* (C29–C34), respectively], with centroid–centroid distances of 3.975 (2) and 3.782 (2) Å, respectively, may further stabilize the structure.

## Synthesis and crystallization   

The title compound was synthesized by the reaction of 5-[(3,5-di­fluoro­phen­yl)diazen­yl]-2-hy­droxy­benzaldehyde (Ba & Ma­thias, 2013 ▸) with 4-amino-2,2,6,6-tetra­methyl­piperidine-1-oxyl (4-amino-TEMPO). 4-amino-TEMPO (171 mg, 1 mmol) in hexane (20 ml) was added to a stirred hexa­ne/CHCl_3_ (1:1) solution (70 ml) of 5-[(3,5-di­fluoro­phen­yl)diazen­yl]-2-hy­droxy­benzaldehyde (262 mg, 1 mmol), and heated at 333 K for 2 h. Then, the reaction mixture was left to slowly cool to room temperature. After one day, orange microcrystals were obtained (yield: 348 mg, 84%). Orange block-shaped crystals, suitable for X-ray analysis, were obtained by recrystallization from methanol/CHCl_3_ (1:1) solution by slow evaporation at room temperature after several days (m.p. 473–475 K).

## Refinement   

Crystal data, data collection and structure refinement details are summarized in Table 3 ▸. Atoms H1 and H3 (for OH) and H13 and H35 (for CH) were located in a difference Fourier map and were refined freely. The other C-bound H atoms were positioned geometrically with C—H = 0.93 Å (for aromatic CH), 0.96 Å (for CH_3_), 0.97 Å (for CH_2_) and 0.98 Å (for CH), and constrained to ride on their parent atoms, with *U*_iso_(H) = *xU*_eq_(C), where *x* = 1.5 for methyl H atoms and *x* = 1.2 for other H atoms.

## Supplementary Material

Crystal structure: contains datablock(s) I, global. DOI: 10.1107/S2056989015012049/xu5856sup1.cif

Structure factors: contains datablock(s) I. DOI: 10.1107/S2056989015012049/xu5856Isup2.hkl

CCDC reference: 1408338

Additional supporting information:  crystallographic information; 3D view; checkCIF report

## Figures and Tables

**Table 1:** Selected geometric parameters (, )

N3C13	1.270(3)	N7C35	1.272(3)
			
C13N3C14	121.6(2)	C35N7C36	117.9(2)
			
C17N4C16C15	33.9(4)	N4C16C15C14	44.0(3)
C16N4C17C18	35.4(4)	C14C18C17N4	46.1(3)
C39N8C38C37	36.8(3)	C40C36C37C38	61.4(3)
C38N8C39C40	34.3(3)	C37C36C40C39	59.0(3)
C18C14C15C16	58.4(3)	N8C38C37C36	48.9(3)
C15C14C18C17	59.1(3)	N8C39C40C36	44.0(3)

**Table 2:** Hydrogen-bond geometry (, )

*D*H*A*	*D*H	H*A*	*D* *A*	*D*H*A*
O1H1N3	1.03(5)	1.66(5)	2.585(3)	147(4)
O3H3N7	0.88(4)	1.85(4)	2.639(3)	148(4)
C13H13O4^i^	0.96(2)	2.44(2)	3.324(3)	154.5(2)
C15H15*A*F1^ii^	0.97	2.43	3.218(3)	138
C30H30O2^iii^	0.93	2.36	3.222(3)	154
C35H35O2^iii^	0.97(2)	2.44(2)	3.318(3)	150.5(2)
C37H37*B*F2	0.97	2.48	3.346(3)	148

Symmetry codes: (i) 
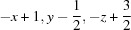
; (ii) 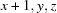; (iii) 
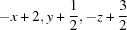
.

**Table 3:** Experimental details

Crystal data	
Chemical formula	C_22_H_25_F_2_N_4_O_2_
*M*_r_	415.46
Crystal system, space group	Monoclinic, *P*2_1_/*c*
Temperature (K)	296
*a*, *b*, *c* ()	13.5115(3), 23.1062(5), 13.8677(3)
()	100.639(3)
*V* (^3^)	4255.06(17)
*Z*	8
Radiation type	Mo *K*
(mm^1^)	0.10
Crystal size (mm)	0.15 0.12 0.07

Data collection	
Diffractometer	Bruker SMART BREEZE CCD
Absorption correction	Multi-scan (*SADABS*; Bruker, 2012 ▸)
*T*_min_, *T*_max_	0.550, 0.746
No. of measured, independent and observed [*I* > 2(*I*)] reflections	73169, 10597, 5159
*R*_int_	0.101
(sin /)_max_ (^1^)	0.669

Refinement	
*R*[*F* ^2^ > 2(*F* ^2^)], *wR*(*F* ^2^), *S*	0.073, 0.163, 1.08
No. of reflections	10597
No. of parameters	565
H-atom treatment	H atoms treated by a mixture of independent and constrained refinement
_max_, _min_ (e ^3^)	0.24, 0.26

Computer programs: *APEX2* and *SAINT* (Bruker, 2012 ▸), *SHELXS97* and *SHELXL97* (Sheldrick, 2008 ▸), *ORTEP-3 for Windows* and *WinGX* (Farrugia, 2012 ▸) and *PLATON* (Spek, 2009 ▸).

## supplementary crystallographic information

### Crystal data

**Table:**

C_22_H_25_F_2_N_4_O_2_	*F*(000) = 1752
*M**_r_* = 415.46	*D*_x_ = 1.297 Mg m^−^^3^
Monoclinic, *P*2_1_/*c*	Mo *K*α radiation, λ = 0.71073 Å
Hall symbol: -P 2ybc	Cell parameters from 9961 reflections
*a* = 13.5115 (3) Å	θ = 3.0–25.5°
*b* = 23.1062 (5) Å	µ = 0.10 mm^−^^1^
*c* = 13.8677 (3) Å	*T* = 296 K
β = 100.639 (3)°	Block, orange
*V* = 4255.06 (17) Å^3^	0.15 × 0.12 × 0.07 mm
*Z* = 8	

### Data collection

**Table:**

Bruker SMART BREEZE CCD diffractometer	10597 independent reflections
Radiation source: fine-focus sealed tube	5159 reflections with *I* > 2σ(*I*)
Graphite monochromator	*R*_int_ = 0.101
φ and ω scans	θ_max_ = 28.4°, θ_min_ = 3.0°
Absorption correction: multi-scan (*SADABS*; Bruker, 2012)	*h* = −18→15
*T*_min_ = 0.550, *T*_max_ = 0.746	*k* = −30→30
73169 measured reflections	*l* = −18→18

### Refinement

**Table:**

Refinement on *F*^2^	Primary atom site location: structure-invariant direct methods
Least-squares matrix: full	Secondary atom site location: difference Fourier map
*R*[*F*^2^ > 2σ(*F*^2^)] = 0.073	Hydrogen site location: inferred from neighbouring sites
*wR*(*F*^2^) = 0.163	H atoms treated by a mixture of independent and constrained refinement
*S* = 1.08	*w* = 1/[σ^2^(*F*_o_^2^) + (0.038*P*)^2^ + 2.7618*P*] where *P* = (*F*_o_^2^ + 2*F*_c_^2^)/3
10597 reflections	(Δ/σ)_max_ < 0.001
565 parameters	Δρ_max_ = 0.24 e Å^−^^3^
0 restraints	Δρ_min_ = −0.26 e Å^−^^3^

### Special details

**Table:**

Geometry. All esds (except the esd in the dihedral angle between two l.s. planes) are estimated using the full covariance matrix. The cell esds are taken into account individually in the estimation of esds in distances, angles and torsion angles; correlations between esds in cell parameters are only used when they are defined by crystal symmetry. An approximate (isotropic) treatment of cell esds is used for estimating esds involving l.s. planes.
Refinement. Refinement of F^2^ against ALL reflections. The weighted R-factor wR and goodness of fit S are based on F^2^, conventional R-factors R are based on F, with F set to zero for negative F^2^. The threshold expression of F^2^ > 2sigma(F^2^) is used only for calculating R-factors(gt) etc. and is not relevant to the choice of reflections for refinement. R-factors based on F^2^ are statistically about twice as large as those based on F, and R- factors based on ALL data will be even larger.

### Fractional atomic coordinates and isotropic or equivalent isotropic displacement parameters (Å^2^)

**Table:**

	*x*	*y*	*z*	*U*_iso_*/*U*_eq_	
O1	1.15807 (13)	0.31895 (8)	0.67257 (16)	0.0571 (6)	
H1	1.182 (3)	0.277 (2)	0.689 (3)	0.147 (17)*	
O2	1.33270 (17)	−0.00335 (9)	0.6821 (2)	0.1014 (10)	
O3	0.35009 (14)	0.31846 (10)	0.84490 (19)	0.0700 (7)	
H3	0.330 (3)	0.3547 (17)	0.837 (3)	0.110 (15)*	
O4	0.10773 (14)	0.61906 (8)	0.74731 (15)	0.0621 (6)	
N1	0.69757 (16)	0.36539 (9)	0.60396 (16)	0.0447 (5)	
N2	0.74088 (15)	0.31783 (9)	0.61875 (16)	0.0436 (5)	
N3	1.14968 (15)	0.20870 (8)	0.70174 (16)	0.0395 (5)	
N4	1.28781 (16)	0.04498 (9)	0.6852 (2)	0.0555 (7)	
N5	0.81042 (17)	0.26830 (10)	0.88903 (17)	0.0496 (6)	
N6	0.76620 (16)	0.31481 (9)	0.87086 (16)	0.0458 (6)	
N7	0.36191 (15)	0.43076 (9)	0.81433 (16)	0.0440 (5)	
N8	0.16577 (15)	0.57475 (9)	0.76354 (16)	0.0413 (5)	
F1	0.38227 (15)	0.26409 (9)	0.5857 (2)	0.1154 (9)	
F2	0.38710 (13)	0.46381 (8)	0.54690 (17)	0.0887 (6)	
F3	1.12098 (14)	0.37687 (9)	0.91031 (17)	0.0940 (7)	
F4	1.12618 (13)	0.17533 (8)	0.93352 (14)	0.0785 (6)	
C1	0.58988 (18)	0.36132 (11)	0.59137 (19)	0.0406 (6)	
C2	0.53935 (19)	0.41297 (12)	0.5745 (2)	0.0476 (7)	
H2	0.5743	0.4474	0.5714	0.057*	
C3	0.4371 (2)	0.41268 (13)	0.5624 (2)	0.0542 (8)	
C4	0.3814 (2)	0.36392 (14)	0.5667 (2)	0.0593 (8)	
H4	0.3115	0.3649	0.5589	0.071*	
C5	0.4345 (2)	0.31360 (14)	0.5831 (3)	0.0634 (9)	
C6	0.5379 (2)	0.31004 (12)	0.5959 (2)	0.0552 (8)	
H6	0.5712	0.2748	0.6071	0.066*	
C7	0.84776 (17)	0.32118 (10)	0.63156 (18)	0.0370 (6)	
C8	0.90275 (19)	0.37183 (11)	0.6247 (2)	0.0457 (7)	
H8	0.8691	0.4068	0.6109	0.055*	
C9	1.00523 (19)	0.37041 (11)	0.6379 (2)	0.0491 (7)	
H9	1.0406	0.4045	0.6329	0.059*	
C10	1.05804 (18)	0.31848 (10)	0.65898 (19)	0.0396 (6)	
C11	1.00326 (17)	0.26725 (10)	0.66530 (17)	0.0326 (5)	
C12	0.89906 (18)	0.26979 (10)	0.65085 (18)	0.0368 (6)	
H12	0.8629	0.2359	0.6543	0.044*	
C13	1.05423 (19)	0.21225 (11)	0.68549 (19)	0.0365 (6)	
H13	1.0106 (17)	0.1798 (10)	0.6857 (17)	0.037 (7)*	
C14	1.20161 (17)	0.15317 (10)	0.72328 (19)	0.0377 (6)	
H14	1.2333	0.1528	0.7928	0.045*	
C15	1.28355 (18)	0.15037 (11)	0.6628 (2)	0.0457 (7)	
H15A	1.3253	0.1846	0.6763	0.055*	
H15B	1.2525	0.1514	0.5939	0.055*	
C16	1.35082 (18)	0.09733 (11)	0.6807 (2)	0.0444 (7)	
C17	1.19288 (19)	0.04364 (11)	0.7258 (2)	0.0475 (7)	
C18	1.13575 (18)	0.09998 (10)	0.7027 (2)	0.0416 (6)	
H18A	1.1040	0.1000	0.6340	0.050*	
H18B	1.0828	0.1020	0.7412	0.050*	
C19	1.4049 (2)	0.08989 (14)	0.5944 (3)	0.0702 (9)	
H19A	1.3563	0.0836	0.5355	0.105*	
H19B	1.4494	0.0572	0.6060	0.105*	
H19C	1.4431	0.1241	0.5872	0.105*	
C20	1.4281 (2)	0.10201 (14)	0.7758 (2)	0.0651 (9)	
H20A	1.4610	0.0654	0.7901	0.098*	
H20B	1.3947	0.1126	0.8285	0.098*	
H20C	1.4772	0.1309	0.7684	0.098*	
C21	1.2180 (2)	0.03272 (14)	0.8364 (3)	0.0711 (10)	
H21A	1.2575	0.0643	0.8679	0.107*	
H21B	1.2555	−0.0026	0.8489	0.107*	
H21C	1.1568	0.0296	0.8618	0.107*	
C22	1.1290 (2)	−0.00633 (12)	0.6769 (3)	0.0732 (10)	
H22A	1.1649	−0.0420	0.6919	0.110*	
H22B	1.1151	−0.0006	0.6072	0.110*	
H22C	1.0668	−0.0078	0.7009	0.110*	
C23	0.91854 (18)	0.27444 (12)	0.89988 (18)	0.0422 (6)	
C24	0.9677 (2)	0.32733 (12)	0.8995 (2)	0.0493 (7)	
H24	0.9322	0.3620	0.8917	0.059*	
C25	1.0708 (2)	0.32625 (13)	0.9110 (2)	0.0552 (8)	
C26	1.1267 (2)	0.27622 (14)	0.9232 (2)	0.0548 (8)	
H26	1.1966	0.2766	0.9310	0.066*	
C27	1.0737 (2)	0.22591 (13)	0.9234 (2)	0.0509 (7)	
C28	0.9715 (2)	0.22356 (12)	0.91185 (19)	0.0464 (7)	
H28	0.9384	0.1883	0.9121	0.056*	
C29	0.65870 (18)	0.31143 (11)	0.86313 (19)	0.0410 (6)	
C30	0.60837 (18)	0.36318 (11)	0.84394 (18)	0.0385 (6)	
H30	0.6449	0.3962	0.8348	0.046*	
C31	0.50519 (17)	0.36772 (10)	0.83778 (18)	0.0364 (6)	
C32	0.45009 (19)	0.31767 (11)	0.8503 (2)	0.0479 (7)	
C33	0.5006 (2)	0.26487 (12)	0.8682 (2)	0.0594 (8)	
H33	0.4645	0.2314	0.8757	0.071*	
C34	0.6032 (2)	0.26191 (12)	0.8748 (2)	0.0558 (8)	
H34	0.6360	0.2265	0.8871	0.067*	
C35	0.45585 (19)	0.42366 (11)	0.81786 (19)	0.0386 (6)	
H35	0.4994 (18)	0.4552 (11)	0.8064 (17)	0.042 (7)*	
C36	0.32030 (18)	0.48883 (10)	0.79230 (19)	0.0399 (6)	
H36	0.3724	0.5145	0.7756	0.048*	
C37	0.23279 (19)	0.48515 (11)	0.7065 (2)	0.0446 (7)	
H37A	0.1816	0.4599	0.7242	0.054*	
H37B	0.2562	0.4679	0.6510	0.054*	
C38	0.18551 (18)	0.54383 (11)	0.67565 (19)	0.0403 (6)	
C39	0.23265 (19)	0.57273 (12)	0.8622 (2)	0.0453 (7)	
C40	0.2808 (2)	0.51305 (12)	0.8792 (2)	0.0498 (7)	
H40A	0.3361	0.5152	0.9347	0.060*	
H40B	0.2314	0.4863	0.8963	0.060*	
C41	0.2535 (2)	0.58093 (13)	0.6231 (2)	0.0565 (8)	
H41A	0.2251	0.6189	0.6115	0.085*	
H41B	0.2587	0.5633	0.5615	0.085*	
H41C	0.3193	0.5838	0.6633	0.085*	
C42	0.0843 (2)	0.53434 (13)	0.6073 (2)	0.0603 (8)	
H42A	0.0554	0.5711	0.5855	0.090*	
H42B	0.0396	0.5141	0.6420	0.090*	
H42C	0.0943	0.5119	0.5517	0.090*	
C43	0.1667 (2)	0.58384 (14)	0.9378 (2)	0.0681 (9)	
H43A	0.1417	0.6228	0.9311	0.102*	
H43B	0.2056	0.5785	1.0024	0.102*	
H43C	0.1111	0.5573	0.9276	0.102*	
C44	0.3117 (2)	0.62071 (13)	0.8681 (2)	0.0645 (9)	
H44A	0.2788	0.6570	0.8503	0.097*	
H44B	0.3566	0.6121	0.8239	0.097*	
H44C	0.3492	0.6231	0.9339	0.097*	

### Atomic displacement parameters (Å^2^)

**Table:**

	*U*^11^	*U*^22^	*U*^33^	*U*^12^	*U*^13^	*U*^23^
O1	0.0320 (10)	0.0402 (11)	0.0952 (17)	−0.0017 (8)	0.0016 (10)	0.0106 (11)
O2	0.0688 (15)	0.0378 (12)	0.210 (3)	0.0163 (11)	0.0583 (18)	−0.0083 (15)
O3	0.0347 (11)	0.0505 (14)	0.124 (2)	0.0017 (10)	0.0142 (11)	0.0242 (13)
O4	0.0562 (12)	0.0607 (13)	0.0693 (14)	0.0315 (10)	0.0110 (10)	0.0061 (11)
N1	0.0413 (12)	0.0410 (13)	0.0513 (15)	0.0057 (10)	0.0071 (10)	0.0037 (11)
N2	0.0415 (12)	0.0403 (13)	0.0482 (14)	0.0103 (10)	0.0064 (10)	0.0032 (10)
N3	0.0353 (12)	0.0284 (11)	0.0544 (14)	0.0073 (9)	0.0070 (10)	0.0030 (10)
N4	0.0425 (13)	0.0309 (12)	0.096 (2)	0.0084 (10)	0.0204 (13)	−0.0069 (12)
N5	0.0479 (13)	0.0463 (14)	0.0537 (15)	0.0086 (11)	0.0073 (11)	0.0015 (11)
N6	0.0426 (13)	0.0445 (14)	0.0483 (14)	0.0113 (11)	0.0031 (10)	−0.0030 (11)
N7	0.0337 (12)	0.0378 (12)	0.0587 (15)	0.0054 (9)	0.0040 (10)	0.0087 (10)
N8	0.0340 (11)	0.0410 (12)	0.0489 (14)	0.0107 (10)	0.0079 (10)	0.0039 (10)
F1	0.0722 (14)	0.0731 (14)	0.202 (3)	−0.0253 (11)	0.0276 (15)	0.0178 (15)
F2	0.0584 (11)	0.0752 (13)	0.1328 (19)	0.0288 (10)	0.0188 (11)	0.0253 (12)
F3	0.0672 (13)	0.0775 (14)	0.1341 (19)	−0.0210 (11)	0.0101 (12)	−0.0166 (13)
F4	0.0702 (12)	0.0810 (13)	0.0842 (14)	0.0358 (10)	0.0138 (10)	0.0009 (11)
C1	0.0326 (13)	0.0491 (16)	0.0398 (16)	0.0013 (12)	0.0053 (11)	0.0009 (12)
C2	0.0402 (15)	0.0459 (16)	0.0566 (18)	0.0028 (12)	0.0089 (13)	0.0054 (13)
C3	0.0427 (16)	0.060 (2)	0.059 (2)	0.0133 (15)	0.0092 (14)	0.0072 (15)
C4	0.0343 (15)	0.073 (2)	0.070 (2)	0.0052 (15)	0.0078 (14)	0.0078 (17)
C5	0.0500 (18)	0.060 (2)	0.081 (2)	−0.0142 (16)	0.0127 (16)	0.0053 (17)
C6	0.0504 (18)	0.0455 (17)	0.069 (2)	0.0062 (14)	0.0094 (15)	0.0054 (15)
C7	0.0332 (13)	0.0385 (14)	0.0379 (15)	0.0063 (11)	0.0034 (11)	0.0007 (11)
C8	0.0448 (15)	0.0291 (14)	0.0585 (18)	0.0106 (12)	−0.0026 (13)	0.0034 (12)
C9	0.0420 (16)	0.0272 (14)	0.073 (2)	−0.0025 (11)	−0.0017 (14)	0.0053 (13)
C10	0.0340 (14)	0.0337 (14)	0.0484 (17)	0.0014 (11)	0.0003 (12)	0.0008 (12)
C11	0.0343 (13)	0.0295 (13)	0.0326 (14)	0.0047 (10)	0.0028 (10)	0.0001 (10)
C12	0.0357 (13)	0.0308 (13)	0.0434 (15)	0.0011 (11)	0.0066 (11)	0.0024 (11)
C13	0.0361 (14)	0.0300 (14)	0.0440 (16)	0.0027 (11)	0.0089 (12)	0.0013 (11)
C14	0.0343 (13)	0.0302 (13)	0.0480 (16)	0.0084 (11)	0.0063 (12)	0.0038 (11)
C15	0.0398 (15)	0.0368 (15)	0.0619 (19)	0.0014 (12)	0.0130 (13)	0.0043 (13)
C16	0.0345 (14)	0.0349 (14)	0.0651 (19)	0.0039 (11)	0.0130 (13)	−0.0025 (13)
C17	0.0381 (15)	0.0299 (14)	0.076 (2)	0.0043 (11)	0.0143 (14)	0.0031 (13)
C18	0.0325 (13)	0.0316 (13)	0.0618 (18)	0.0054 (11)	0.0113 (12)	0.0047 (12)
C19	0.0532 (19)	0.073 (2)	0.091 (3)	0.0033 (16)	0.0298 (18)	−0.0120 (19)
C20	0.0453 (17)	0.064 (2)	0.082 (2)	0.0101 (15)	0.0013 (16)	−0.0037 (17)
C21	0.068 (2)	0.058 (2)	0.088 (3)	0.0116 (16)	0.0157 (19)	0.0267 (18)
C22	0.061 (2)	0.0371 (17)	0.125 (3)	−0.0043 (15)	0.025 (2)	−0.0098 (18)
C23	0.0338 (14)	0.0590 (18)	0.0333 (15)	0.0040 (13)	0.0044 (11)	−0.0050 (13)
C24	0.0477 (17)	0.0505 (17)	0.0482 (18)	0.0114 (13)	0.0053 (13)	−0.0076 (13)
C25	0.0466 (17)	0.0594 (19)	0.058 (2)	−0.0081 (15)	0.0056 (14)	−0.0132 (15)
C26	0.0318 (14)	0.080 (2)	0.0496 (18)	0.0062 (15)	0.0013 (13)	−0.0148 (16)
C27	0.0465 (17)	0.068 (2)	0.0379 (16)	0.0199 (15)	0.0064 (13)	−0.0033 (14)
C28	0.0475 (16)	0.0498 (17)	0.0412 (16)	0.0055 (13)	0.0067 (13)	−0.0011 (13)
C29	0.0340 (14)	0.0429 (15)	0.0445 (16)	0.0069 (12)	0.0028 (12)	0.0002 (12)
C30	0.0336 (13)	0.0372 (14)	0.0432 (16)	−0.0006 (11)	0.0031 (11)	0.0018 (12)
C31	0.0316 (13)	0.0345 (14)	0.0414 (15)	0.0048 (11)	0.0026 (11)	0.0030 (11)
C32	0.0341 (15)	0.0428 (16)	0.065 (2)	0.0024 (12)	0.0056 (13)	0.0085 (14)
C33	0.0492 (18)	0.0355 (16)	0.093 (2)	0.0006 (13)	0.0113 (16)	0.0163 (15)
C34	0.0498 (17)	0.0384 (16)	0.078 (2)	0.0133 (13)	0.0080 (15)	0.0102 (15)
C35	0.0346 (14)	0.0349 (14)	0.0450 (16)	0.0011 (12)	0.0036 (12)	0.0031 (12)
C36	0.0332 (13)	0.0321 (14)	0.0529 (17)	0.0047 (11)	0.0040 (12)	0.0066 (12)
C37	0.0415 (15)	0.0403 (15)	0.0508 (17)	0.0056 (12)	0.0055 (13)	−0.0010 (13)
C38	0.0359 (14)	0.0439 (15)	0.0400 (15)	0.0070 (11)	0.0039 (11)	0.0020 (12)
C39	0.0427 (15)	0.0477 (16)	0.0438 (16)	0.0106 (12)	0.0037 (12)	−0.0016 (13)
C40	0.0493 (16)	0.0521 (17)	0.0442 (17)	0.0091 (13)	−0.0009 (13)	0.0049 (13)
C41	0.0529 (17)	0.0591 (19)	0.060 (2)	0.0108 (14)	0.0168 (15)	0.0153 (15)
C42	0.0532 (18)	0.0578 (19)	0.062 (2)	0.0071 (15)	−0.0084 (15)	0.0037 (15)
C43	0.074 (2)	0.076 (2)	0.057 (2)	0.0174 (18)	0.0183 (17)	−0.0042 (17)
C44	0.0556 (19)	0.0546 (19)	0.079 (2)	0.0012 (15)	0.0027 (17)	−0.0122 (17)

### Geometric parameters (Å, º)

**Table:**

O1—C10	1.330 (3)	C19—H19A	0.9600
O1—H1	1.03 (5)	C19—H19B	0.9600
O3—C32	1.339 (3)	C19—H19C	0.9600
O3—H3	0.88 (4)	C20—H20A	0.9600
O4—N8	1.284 (2)	C20—H20B	0.9600
N1—C1	1.436 (3)	C20—H20C	0.9600
N2—N1	1.244 (3)	C21—H21A	0.9600
N2—C7	1.424 (3)	C21—H21B	0.9600
N3—C13	1.270 (3)	C21—H21C	0.9600
N3—C14	1.466 (3)	C22—H22A	0.9600
N4—O2	1.275 (3)	C22—H22B	0.9600
N4—C16	1.487 (3)	C22—H22C	0.9600
N4—C17	1.493 (3)	C23—C24	1.392 (4)
N5—C23	1.448 (3)	C23—C28	1.370 (4)
N6—N5	1.233 (3)	C24—C25	1.373 (4)
N6—C29	1.439 (3)	C24—H24	0.9300
N7—C35	1.272 (3)	C26—C25	1.374 (4)
N7—C36	1.465 (3)	C26—H26	0.9300
N8—C38	1.479 (3)	C27—C26	1.365 (4)
N8—C39	1.495 (3)	C28—C27	1.362 (4)
F1—C5	1.348 (3)	C28—H28	0.9300
F2—C3	1.358 (3)	C29—C34	1.393 (4)
F3—C25	1.353 (3)	C30—C29	1.377 (3)
F4—C27	1.360 (3)	C30—H30	0.9300
C1—C2	1.373 (3)	C31—C30	1.385 (3)
C1—C6	1.385 (4)	C31—C32	1.403 (3)
C2—C3	1.360 (4)	C32—C33	1.398 (4)
C2—H2	0.9300	C33—H33	0.9300
C3—C4	1.363 (4)	C34—C33	1.374 (4)
C4—C5	1.363 (4)	C34—H34	0.9300
C4—H4	0.9300	C35—C31	1.457 (3)
C6—C5	1.377 (4)	C35—H35	0.97 (2)
C6—H6	0.9300	C36—C37	1.517 (3)
C7—C8	1.399 (3)	C36—C40	1.512 (4)
C8—C9	1.363 (3)	C36—H36	0.9800
C8—H8	0.9300	C37—H37A	0.9700
C9—H9	0.9300	C37—H37B	0.9700
C10—C9	1.399 (3)	C38—C37	1.526 (3)
C11—C10	1.408 (3)	C38—C41	1.536 (4)
C11—C12	1.386 (3)	C38—C42	1.529 (4)
C11—C13	1.448 (3)	C39—C40	1.525 (3)
C12—C7	1.376 (3)	C39—C43	1.517 (4)
C12—H12	0.9300	C39—C44	1.531 (4)
C13—H13	0.96 (2)	C40—H40A	0.9700
C14—C15	1.509 (3)	C40—H40B	0.9700
C14—C18	1.513 (3)	C41—H41A	0.9600
C14—H14	0.9800	C41—H41B	0.9600
C15—H15A	0.9700	C41—H41C	0.9600
C15—H15B	0.9700	C42—H42A	0.9600
C16—C15	1.519 (3)	C42—H42B	0.9600
C16—C19	1.523 (4)	C42—H42C	0.9600
C16—C20	1.528 (4)	C43—H43A	0.9600
C17—C21	1.528 (4)	C43—H43B	0.9600
C17—C22	1.524 (4)	C43—H43C	0.9600
C18—C17	1.517 (3)	C44—H44A	0.9600
C18—H18A	0.9700	C44—H44B	0.9600
C18—H18B	0.9700	C44—H44C	0.9600
			
C10—O1—H1	107 (2)	H21B—C21—H21C	109.5
C32—O3—H3	108 (3)	C17—C22—H22A	109.5
N2—N1—C1	113.4 (2)	C17—C22—H22B	109.5
N1—N2—C7	114.1 (2)	C17—C22—H22C	109.5
C13—N3—C14	121.6 (2)	H22A—C22—H22B	109.5
O2—N4—C16	115.5 (2)	H22A—C22—H22C	109.5
O2—N4—C17	116.2 (2)	H22B—C22—H22C	109.5
C16—N4—C17	124.8 (2)	C24—C23—N5	124.0 (2)
N6—N5—C23	112.2 (2)	C28—C23—N5	115.0 (2)
N5—N6—C29	114.4 (2)	C28—C23—C24	121.0 (2)
C35—N7—C36	117.9 (2)	C23—C24—H24	121.4
O4—N8—C38	116.0 (2)	C25—C24—C23	117.3 (3)
O4—N8—C39	115.6 (2)	C25—C24—H24	121.4
C38—N8—C39	124.67 (19)	F3—C25—C24	118.8 (3)
C2—C1—N1	115.1 (2)	F3—C25—C26	117.6 (3)
C2—C1—C6	120.7 (2)	C24—C25—C26	123.5 (3)
C6—C1—N1	124.2 (2)	C25—C26—H26	121.9
C1—C2—H2	120.7	C27—C26—C25	116.1 (3)
C3—C2—C1	118.6 (3)	C27—C26—H26	122.0
C3—C2—H2	120.7	F4—C27—C28	118.4 (3)
F2—C3—C2	118.7 (3)	F4—C27—C26	117.9 (3)
F2—C3—C4	117.6 (3)	C28—C27—C26	123.7 (3)
C2—C3—C4	123.8 (3)	C23—C28—H28	120.8
C3—C4—C5	115.7 (3)	C27—C28—C23	118.4 (3)
C3—C4—H4	122.1	C27—C28—H28	120.8
C5—C4—H4	122.1	C30—C29—N6	115.0 (2)
F1—C5—C4	117.8 (3)	C30—C29—C34	118.6 (2)
F1—C5—C6	118.0 (3)	C34—C29—N6	126.4 (2)
C4—C5—C6	124.2 (3)	C29—C30—C31	122.2 (2)
C1—C6—H6	121.5	C29—C30—H30	118.9
C5—C6—C1	117.0 (3)	C31—C30—H30	118.9
C5—C6—H6	121.5	C30—C31—C32	118.7 (2)
C8—C7—N2	125.2 (2)	C30—C31—C35	119.9 (2)
C12—C7—N2	116.1 (2)	C32—C31—C35	121.3 (2)
C12—C7—C8	118.7 (2)	O3—C32—C31	122.3 (2)
C7—C8—H8	119.7	O3—C32—C33	118.5 (2)
C9—C8—C7	120.6 (2)	C33—C32—C31	119.2 (2)
C9—C8—H8	119.7	C32—C33—H33	119.7
C8—C9—C10	121.0 (2)	C34—C33—C32	120.6 (3)
C8—C9—H9	119.5	C34—C33—H33	119.7
C10—C9—H9	119.5	C29—C34—H34	119.7
O1—C10—C9	119.2 (2)	C33—C34—C29	120.6 (2)
O1—C10—C11	122.0 (2)	C33—C34—H34	119.7
C9—C10—C11	118.7 (2)	N7—C35—C31	122.7 (2)
C10—C11—C13	120.9 (2)	N7—C35—H35	122.0 (14)
C12—C11—C10	119.1 (2)	C31—C35—H35	115.3 (14)
C12—C11—C13	120.0 (2)	N7—C36—C40	110.6 (2)
C7—C12—C11	121.8 (2)	N7—C36—C37	109.0 (2)
C7—C12—H12	119.1	N7—C36—H36	109.8
C11—C12—H12	119.1	C37—C36—H36	109.8
N3—C13—C11	121.5 (2)	C40—C36—C37	107.9 (2)
N3—C13—H13	123.8 (14)	C40—C36—H36	109.8
C11—C13—H13	114.7 (14)	C36—C37—C38	113.3 (2)
N3—C14—C15	107.3 (2)	C36—C37—H37A	108.9
N3—C14—C18	115.44 (19)	C36—C37—H37B	108.9
N3—C14—H14	108.3	C38—C37—H37A	108.9
C15—C14—C18	109.0 (2)	C38—C37—H37B	108.9
C15—C14—H14	108.3	H37A—C37—H37B	107.7
C18—C14—H14	108.3	N8—C38—C37	109.2 (2)
C14—C15—C16	115.0 (2)	N8—C38—C42	107.6 (2)
C14—C15—H15A	108.5	N8—C38—C41	109.7 (2)
C14—C15—H15B	108.5	C37—C38—C42	109.1 (2)
C16—C15—H15A	108.5	C37—C38—C41	111.9 (2)
C16—C15—H15B	108.5	C42—C38—C41	109.3 (2)
H15A—C15—H15B	107.5	N8—C39—C43	107.1 (2)
N4—C16—C15	109.5 (2)	N8—C39—C40	109.7 (2)
N4—C16—C19	107.7 (2)	N8—C39—C44	108.9 (2)
N4—C16—C20	109.0 (2)	C43—C39—C40	109.6 (2)
C15—C16—C19	108.8 (2)	C43—C39—C44	109.5 (2)
C15—C16—C20	112.1 (2)	C40—C39—C44	111.9 (2)
C19—C16—C20	109.6 (2)	C36—C40—C39	114.6 (2)
N4—C17—C18	109.9 (2)	C36—C40—H40A	108.6
N4—C17—C22	107.7 (2)	C36—C40—H40B	108.6
N4—C17—C21	109.5 (2)	C39—C40—H40A	108.6
C18—C17—C22	109.4 (2)	C39—C40—H40B	108.6
C18—C17—C21	111.4 (2)	H40A—C40—H40B	107.6
C22—C17—C21	108.9 (2)	C38—C41—H41A	109.5
C14—C18—C17	113.5 (2)	C38—C41—H41B	109.5
C14—C18—H18A	108.9	C38—C41—H41C	109.5
C14—C18—H18B	108.9	H41A—C41—H41B	109.5
C17—C18—H18A	108.9	H41A—C41—H41C	109.5
C17—C18—H18B	108.9	H41B—C41—H41C	109.5
H18A—C18—H18B	107.7	C38—C42—H42A	109.5
C16—C19—H19A	109.5	C38—C42—H42B	109.5
C16—C19—H19B	109.5	C38—C42—H42C	109.5
C16—C19—H19C	109.5	H42A—C42—H42B	109.5
H19A—C19—H19B	109.5	H42A—C42—H42C	109.5
H19A—C19—H19C	109.5	H42B—C42—H42C	109.5
H19B—C19—H19C	109.5	C39—C43—H43A	109.5
C16—C20—H20A	109.5	C39—C43—H43B	109.5
C16—C20—H20B	109.5	C39—C43—H43C	109.5
C16—C20—H20C	109.5	H43A—C43—H43B	109.5
H20A—C20—H20B	109.5	H43A—C43—H43C	109.5
H20A—C20—H20C	109.5	H43B—C43—H43C	109.5
H20B—C20—H20C	109.5	C39—C44—H44A	109.5
C17—C21—H21A	109.5	C39—C44—H44B	109.5
C17—C21—H21B	109.5	C39—C44—H44C	109.5
C17—C21—H21C	109.5	H44A—C44—H44B	109.5
H21A—C21—H21B	109.5	H44A—C44—H44C	109.5
H21A—C21—H21C	109.5	H44B—C44—H44C	109.5
			
N2—N1—C1—C2	−179.8 (2)	C12—C11—C10—O1	179.7 (2)
N2—N1—C1—C6	0.6 (4)	C12—C11—C10—C9	−0.2 (4)
C7—N2—N1—C1	−179.9 (2)	C13—C11—C10—O1	−1.0 (4)
N1—N2—C7—C8	−2.7 (4)	C13—C11—C10—C9	179.1 (2)
N1—N2—C7—C12	178.3 (2)	C10—C11—C12—C7	−0.7 (4)
C14—N3—C13—C11	179.3 (2)	C13—C11—C12—C7	180.0 (2)
C13—N3—C14—C15	134.9 (3)	C10—C11—C13—N3	2.7 (4)
C13—N3—C14—C18	13.1 (4)	C12—C11—C13—N3	−178.0 (2)
O2—N4—C16—C15	167.8 (3)	C11—C12—C7—N2	−179.6 (2)
O2—N4—C16—C19	49.6 (3)	C11—C12—C7—C8	1.2 (4)
O2—N4—C16—C20	−69.2 (3)	N3—C14—C15—C16	175.9 (2)
C17—N4—C16—C15	−33.9 (4)	C18—C14—C15—C16	−58.4 (3)
C17—N4—C16—C19	−152.1 (3)	N3—C14—C18—C17	179.9 (2)
C17—N4—C16—C20	89.1 (3)	C15—C14—C18—C17	59.1 (3)
O2—N4—C17—C18	−166.4 (3)	N4—C16—C15—C14	44.0 (3)
O2—N4—C17—C21	70.9 (3)	C19—C16—C15—C14	161.5 (2)
O2—N4—C17—C22	−47.4 (4)	C20—C16—C15—C14	−77.1 (3)
C16—N4—C17—C18	35.4 (4)	C14—C18—C17—N4	−46.1 (3)
C16—N4—C17—C21	−87.3 (3)	C14—C18—C17—C21	75.5 (3)
C16—N4—C17—C22	154.5 (3)	C14—C18—C17—C22	−164.1 (2)
N6—N5—C23—C24	5.9 (4)	N5—C23—C24—C25	179.7 (2)
N6—N5—C23—C28	−174.7 (2)	C28—C23—C24—C25	0.3 (4)
C29—N6—N5—C23	−177.7 (2)	N5—C23—C28—C27	−179.5 (2)
N5—N6—C29—C30	179.3 (2)	C24—C23—C28—C27	0.0 (4)
N5—N6—C29—C34	0.5 (4)	C23—C24—C25—F3	179.6 (3)
C36—N7—C35—C31	−179.1 (2)	C23—C24—C25—C26	−0.3 (4)
C35—N7—C36—C37	126.6 (3)	C27—C26—C25—F3	−179.9 (3)
C35—N7—C36—C40	−115.0 (3)	C27—C26—C25—C24	0.1 (4)
O4—N8—C38—C37	−166.1 (2)	F4—C27—C26—C25	178.9 (2)
O4—N8—C38—C41	71.0 (3)	C28—C27—C26—C25	0.3 (4)
O4—N8—C38—C42	−47.9 (3)	C23—C28—C27—F4	−178.9 (2)
C39—N8—C38—C37	36.8 (3)	C23—C28—C27—C26	−0.3 (4)
C39—N8—C38—C41	−86.1 (3)	N6—C29—C34—C33	178.2 (3)
C39—N8—C38—C42	155.1 (2)	C30—C29—C34—C33	−0.6 (4)
O4—N8—C39—C40	168.5 (2)	C31—C30—C29—N6	−177.7 (2)
O4—N8—C39—C43	49.6 (3)	C31—C30—C29—C34	1.3 (4)
O4—N8—C39—C44	−68.6 (3)	C32—C31—C30—C29	−0.8 (4)
C38—N8—C39—C40	−34.3 (3)	C35—C31—C30—C29	179.6 (2)
C38—N8—C39—C43	−153.2 (2)	C30—C31—C32—O3	−179.6 (3)
C38—N8—C39—C44	88.5 (3)	C30—C31—C32—C33	−0.2 (4)
N1—C1—C2—C3	−179.6 (2)	C35—C31—C32—O3	−0.1 (4)
C6—C1—C2—C3	0.1 (4)	C35—C31—C32—C33	179.3 (3)
N1—C1—C6—C5	179.4 (3)	O3—C32—C33—C34	−179.7 (3)
C2—C1—C6—C5	−0.2 (4)	C31—C32—C33—C34	0.9 (5)
C1—C2—C3—F2	179.2 (3)	C29—C34—C33—C32	−0.4 (5)
C1—C2—C3—C4	0.4 (5)	N7—C35—C31—C30	−177.8 (3)
F2—C3—C4—C5	−179.5 (3)	N7—C35—C31—C32	2.7 (4)
C2—C3—C4—C5	−0.7 (5)	N7—C36—C37—C38	−178.5 (2)
C3—C4—C5—F1	−178.6 (3)	C40—C36—C37—C38	61.4 (3)
C3—C4—C5—C6	0.6 (5)	N7—C36—C40—C39	−178.1 (2)
C1—C6—C5—F1	179.0 (3)	C37—C36—C40—C39	−59.0 (3)
C1—C6—C5—C4	−0.1 (5)	N8—C38—C37—C36	−48.9 (3)
N2—C7—C8—C9	−179.8 (3)	C41—C38—C37—C36	72.6 (3)
C12—C7—C8—C9	−0.8 (4)	C42—C38—C37—C36	−166.3 (2)
C7—C8—C9—C10	−0.2 (4)	N8—C39—C40—C36	44.0 (3)
O1—C10—C9—C8	−179.2 (3)	C43—C39—C40—C36	161.4 (2)
C11—C10—C9—C8	0.7 (4)	C44—C39—C40—C36	−77.0 (3)

### Hydrogen-bond geometry (Å, º)

**Table:**

*D*—H···*A*	*D*—H	H···*A*	*D*···*A*	*D*—H···*A*
O1—H1···N3	1.03 (5)	1.66 (5)	2.585 (3)	147 (4)
O3—H3···N7	0.88 (4)	1.85 (4)	2.639 (3)	148 (4)
C13—H13···O4^i^	0.96 (2)	2.44 (2)	3.324 (3)	154.5 (2)
C15—H15*A*···F1^ii^	0.97	2.43	3.218 (3)	138
C30—H30···O2^iii^	0.93	2.36	3.222 (3)	154
C35—H35···O2^iii^	0.97 (2)	2.44 (2)	3.318 (3)	150.5 (2)
C37—H37*B*···F2	0.97	2.48	3.346 (3)	148

Symmetry codes: (i) −*x*+1, *y*−1/2, −*z*+3/2; (ii) *x*+1, *y*, *z*; (iii) −*x*+2, *y*+1/2, −*z*+3/2.
